# Give What You Get: Capuchin Monkeys (*Cebus apella*) and 4-Year-Old Children Pay Forward Positive and Negative Outcomes to Conspecifics

**DOI:** 10.1371/journal.pone.0087035

**Published:** 2014-01-29

**Authors:** Kristin L. Leimgruber, Adrian F. Ward, Jane Widness, Michael I. Norton, Kristina R. Olson, Kurt Gray, Laurie R. Santos

**Affiliations:** 1 Department of Psychology, Yale University, New Haven, Connecticut, United States of America; 2 Department of Psychology, Harvard University, Cambridge, Massachusetts, United States of America; 3 Harvard Business School, Harvard University, Boston, Massachusetts, United States of America; 4 Department of Psychology, University of Maryland, College Park, Maryland, United States of America; University of Florence, Italy

## Abstract

The breadth of human generosity is unparalleled in the natural world, and much research has explored the mechanisms underlying and motivating human prosocial behavior. Recent work has focused on the spread of prosocial behavior within groups through paying-it-forward, a case of human prosociality in which a recipient of generosity pays a good deed *forward* to a third individual, rather than *back* to the original source of generosity. While research shows that human adults do indeed pay forward generosity, little is known about the origins of this behavior. Here, we show that both capuchin monkeys (*Cebus apella*) and 4-year-old children pay forward positive *and* negative outcomes in an identical testing paradigm. These results suggest that a cognitively simple mechanism present early in phylogeny and ontogeny leads to paying forward positive, as well as negative, outcomes.

## Introduction

Humans frequently and willingly engage in costly behaviors that benefit others, even when their actions are anonymous and when those helped are total strangers [1], [2]. This proclivity for prosocial behavior is unparalleled in the natural world and is thus thought to play a key role in large-scale cooperation unique to human society [3]. As a result, much research has been devoted to understanding the social, cognitive, and biological processes that encourage (and discourage) prosociality in adult humans [4]–[7]. Recently, researchers have begun to explore the spread of prosocial behavior within populations [8], [9]; specifically, several studies have examined when and why people pay forward prosocial behavior [10], [11]. This concept of “paying-it-forward” is simple: Person A helps Person B and Person B, rather than paying this kindness *back* to Person A, pays it *forward* to Person C, thus facilitating the spread of prosocial behavior beyond the dyad to a larger group of individuals. While experimental research [9], [12], [13] and real-life accounts [14] indicate that humans do pay forward positive outcomes, the psychological underpinnings of such behavior remain unresolved. Traditional explanations for paying forward positive outcomes tend to rely on socially and cognitively complex mechanisms including gratitude [15]–[18], cultural and moral norms [19], [20], and processes requiring sophisticated perspective-taking abilities [21]. Taken together, these social and cognitive constraints might suggest that paying forward generosity is a uniquely human phenomenon.

However, a comprehensive review of the existing literature suggests that the tendency to pay-it-forward may instead be explained by more rudimentary behavioral strategies that are not, in fact, unique to human adults. Specifically, it is possible that people act on the basis of the maxim: “help anyone, if helped by someone” (hereafter, help-if-helped) [22]. Unlike more cognitively complex explanations for the propagation of prosocial behavior, this strategy does not require memory of the identities of interaction partners [23], sensitivity to one’s own reputational status [24], [25], the capacity to calculate the potential costs and benefits of prosocial behavior [26], or the use of self-control to inhibit initial selfish urges [27], [28]; instead, the strategy simply requires that individuals do to others what was done to them. Both mathematical models [29], [30] and laboratory simulations [12], [13], [31] have demonstrated that a simple rule like help-if-helped could lead to self-sustaining pay-it-forward systems. Moreover, experiments indicating that rats (*Rattus norvegicus*) pay forward helping behaviors [32] provide further evidence that complex and/or uniquely human social and cognitive capacities are not required for organisms to pay forward generosity. Indeed, these findings show that a help-if-helped strategy is not only sufficient to support the propagation of prosocial behavior within populations, but also that it likely predates more discriminating forms of cooperative behavior that rely upon the complex social and cognitive abilities found only in human adults.

Furthermore, the majority of existing studies investigating the psychology of paying-it-forward focus exclusively on the prosocial side of paying behavior forward–that is, on paying forward *positive* outcomes. However, laboratory simulations of pay-it-forward behavior suggest that negative outcomes are just as likely to be paid forward in public goods games as positive ones [9], and experimental evidence suggests that–in some situations–adults pay forward greed *more* than generosity [10]. These findings, along with a long history of literature on displaced aggression [33], call into question the proposed role of prosocial intentions, positive emotions and moral norms in paying forward like outcomes. Instead, they suggest the existence of a strategy even more simple than help-if-helped: they suggest that pay-it-forward behavior may be based on the rudimentary rule of “give what you get” (hereafter, give-what-you-get).

Taken as a whole, this set of findings hints that existing research–which typically divides paying-it-forward into separate positive and negative phenomena–may be neglecting a more parsimonious explanation for the propagation of behavior in general. While some accounts of paying it forward favor cognitively and morally rich accounts of human kindness [34], [35], empirical evidence suggests that these behaviors may instead be rooted in a general tendency to reciprocate both positive and negative behaviors in kind [9]. If this simple explanation holds true, we would expect to see behaviors consistent with a give-what-you-get mechanism present early in human development, and possibly even in non-human primates.

The current study tests this possibility by examining pay-it-forward tendencies in 4-year-old children and capuchin monkeys (*Cebus apella*). While there is evidence that capuchin monkeys [36]–[38] and young children [39]–[42] consistently take advantage of no-cost opportunities to act prosocially toward conspecifics, both groups lack certain capacities key to current explanations of paying-it-forward in human adults. Specifically, capuchin monkeys largely fail at tasks that rely on perspective-taking abilities [43]–[47], self-awareness [48], [49], and the ability to evaluate and reflect upon their own knowledge states [50], [51] – all cognitive capacities assumed necessary for the experience of gratitude [52]–[54] and implicated in current explanations for paying forward generosity. Similarly, before the age of five, children have difficulty in evaluating the perspectives and knowledge states of others in a consistent manner [55]–[58] and in evaluating and reflecting upon their *own* thoughts and knowledge states [59]. Unlike capuchin monkeys, however, young children have likely been exposed to social and moral norms advocating paying forward generosity in some form or another. Testing these populations using an identical paradigm allows us to identify the minimal cognitive abilities required to pay-it-forward and illuminates the role uniquely human social and moral norms play in the propagation of paying forward generosity.

Participants in the current study took part in a chain of non-anonymous donation games in which individuals first received a positive or negative outcome from a member of their social group, and then had the chance to distribute a positive or negative outcome to a different member of this social group. We used only “no-cost” options, in which participants making donation decisions received the same outcome regardless of the outcome they chose to deliver to a group member. The use of a “no-cost,” (or non-zero-sum) paradigm reduces the role of self-interested motivations, accounts for between-species differences in self-control and/or reputational concerns, and minimizes cognitive demands imposed by trade-off related calculations. Controlling for these factors allowed us to explore the minimal social and cognitive factors underlying pay-it-forward strategies, thus making it possible to identify the most parsimonious explanation for the donation behaviors observed in monkeys and children.

## Materials and Methods

### Ethics Statement

This study was carried out in strict accordance with the recommendations in the Guide for Care and Use of Laboratory Animals of the National Institutes of Health. The protocol for non-human primates was approved by the Institutional Animal Care and Use Committee at Yale University (Protocol Number: #2008-10678). The treatment of human participants in studies described in this paper was in accordance with the ethical standards of the American Psychological Association. Participants’ parents provided written informed consent and all procedures were approved by the Human Research Protection Program at Yale University.

### Participants

Monkey participants were 4 brown capuchins (*Cebus apella*) ranging in age from 5–15 years at the conclusion of the study (1 male [NN], 3 females [HG, HR, JM]; *M_age_* = 134.86 months; *SD = *55.05). Our capuchin participants were members of the Yale Comparative Cognition Laboratory colony where they were socially housed in a large indoor enclosure equipped with natural branches and toys. Capuchins were fed monkey chow prior to testing and had access to water ad libitum. All participants had previous experience with reward distribution tasks involving conspecifics [36] and were familiar with one another prior to testing. To control for the effects of previous experience and developmental differences in social cognitive abilities, only mature adult monkeys who had previously demonstrated an understanding of the apparatus (Unpublished data) were involved in the current study. Although these strict selection criteria limited the number of monkeys we were able to include in the study, our final sample size is nonetheless comparable to those in other studies of social cognition in brown capuchin monkeys [38], [60]–[62].

We also tested 31 four-year-old children (10 males, 21 females; *M_age_* = 54.68 months; *SD = *3.45) recruited from preschools in the greater New England area. Participants were tested in mixed gender groups comprised of children from the same classroom; as a result, all children were familiar with one another prior to testing. Care was taken to ensure that children never received from, or gave to, members of their own family. Group size was constrained by the number of consenting participants per class, with groups ranging in size from 3–7 individuals.

### General Methods

**Figure 1:**
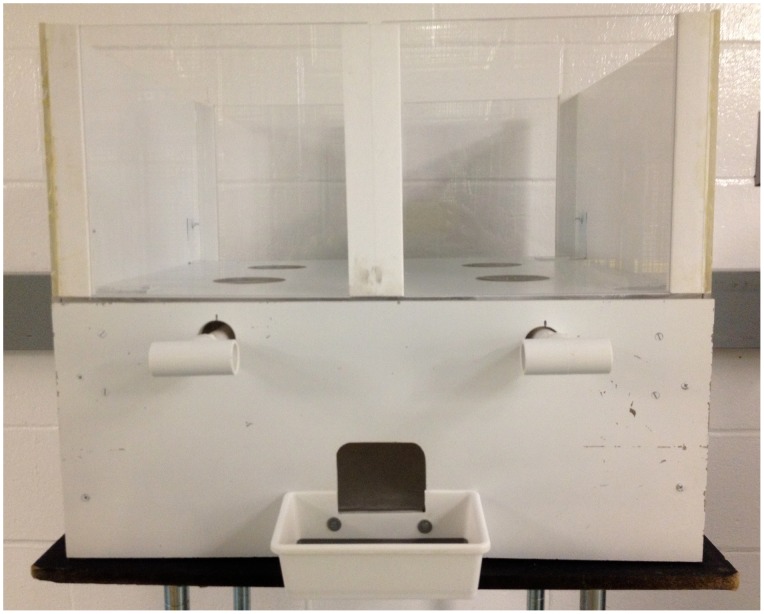
Testing apparatus used for monkeys. Monkey Actors pulled one of the two levers to choose an outcome to distribute to the Receiver situated on the other side of the apparatus.

**Figure 2:**
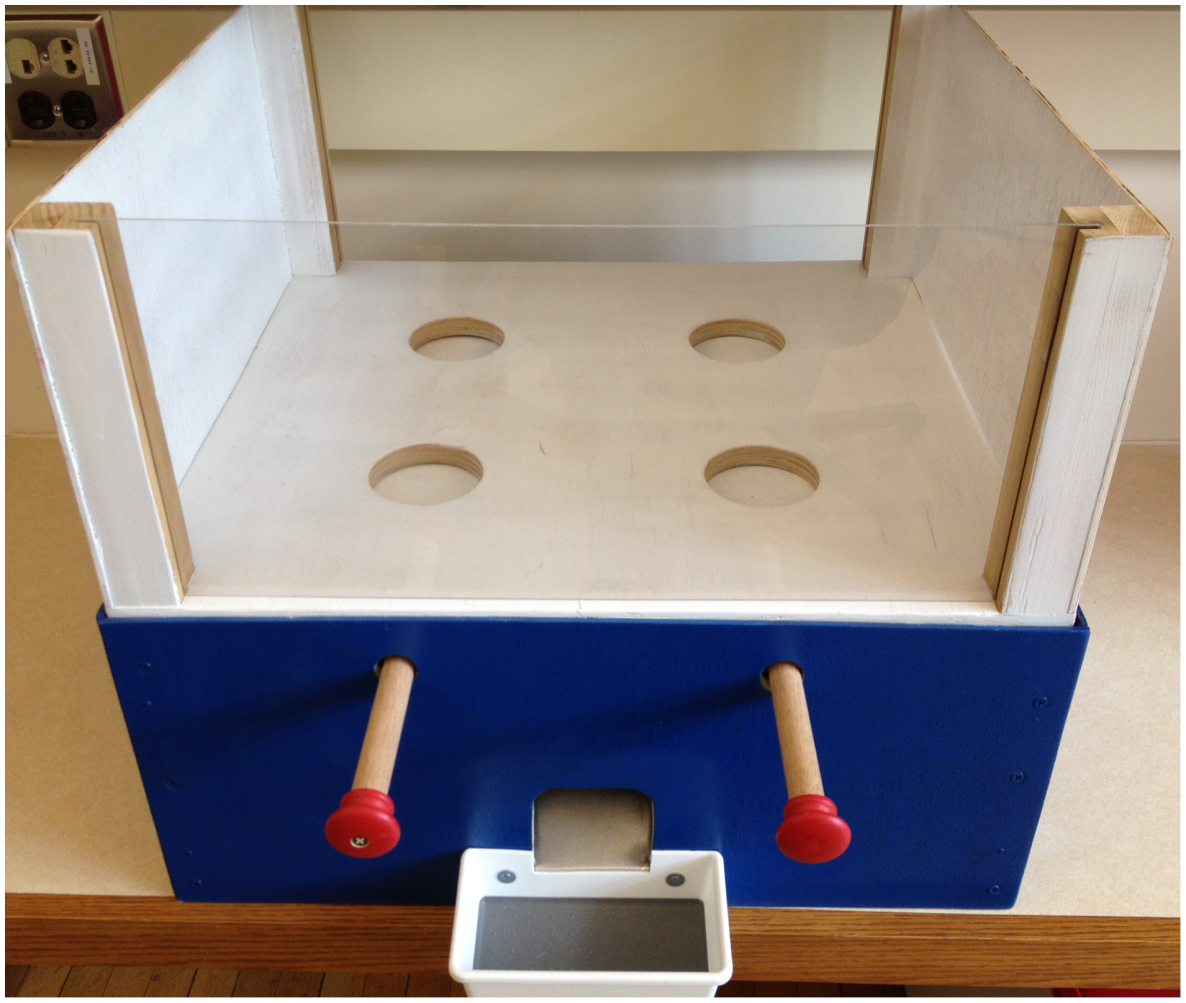
Testing apparatus used for children. Actors pulled one of the two levers to choose an outcome to distribute to the Receiver situated on the other side of the apparatus.

Testing was performed using identical novel apparatuses for the monkeys (Figure 1) and the children (Figure 2) that allowed participants to choose between two distinct distributions. Each distribution provided an allocation for an Actor (the participant manipulating the apparatus), and an allocation for a Recipient (a second participant who merely received whatever he/she was given). The apparatus was situated between the Actor and the Recipient such that the two participants were able to see one another and the distribution options over the top of the apparatus. In order to equate the non-verbal methods as closely as possible across the two populations, children were asked not to speak to one another or signal their preferences in any way. The Actor was always the participant seated on the side of the apparatus with two identical levers. By pulling the lever on her left, the Actor could distribute the leftmost allocations to herself and the Recipient; by pulling the lever on her right, the Actor could distribute the rightmost allocations to herself and the Recipient. Allocations were simultaneously delivered via a chute to both participants immediately following the Actor’s choice, and the two remaining allocations were removed from the apparatus by the experimenter.

Using this apparatus, monkeys and children participated in a series of overlapping donation games, such that each participant in a chain first received from, and then gave to different conspecific members of their social groups. Test sessions began when an initial Actor distributed an outcome to a conspecific Recipient. After both participants had collected their respective allocations, the Recipient moved to the opposite side of the apparatus to assume the role of Actor and the previous Actor left the testing area. At this point, a third conspecific–ignorant to the outcome of the previous interaction–entered the testing area to assume the role of Recipient, and the new Actor was asked to choose between the same distribution options presented to the previous Actor. This process continued until all participants had received from, and subsequently given to, a conspecific group member, with the initial Actor serving as the recipient for the final participant. Data from the initial Actor was excluded from analysis. Efforts were made to ensure that testing and data collection procedures were identical between species whenever possible (however, see Methods S1, Table S1, and Table S2 for between-species methodological differences).

Allocations were placed inside of clear, round, plastic containers that allowed for easy distribution via the apparatus. The placement of the distribution options (positive/negative) into the apparatus was counterbalanced to control for the possible role of side biases in participant’s donation choices (see Methods S1, Table S1, and Table S2 for more details). For each test trial, Actors had the option to deliver one of two outcomes to the Recipient: a positive outcome that delivered a high-value allocation to both herself and the recipient, or a negative outcome that delivered a high-value allocation to herself and a low-value allocation to the Recipient. A positive outcome for monkeys consisted of a grape for both the Actor and the Recipient; a negative outcome consisted of a grape for the Actor and a piece of spinach for the Recipient. A positive outcome for children consisted of 4 small, star-shaped stickers for both the Actor and Recipient; a negative outcome consisted of 4 small, star-shaped stickers for the Actor and 1 small, star-shaped sticker for the Recipient. Actors always received the high-value reward, regardless of what they chose to distribute to Receivers; thus, there was no cost to generosity and no benefit from greed–participants’ distributions to conspecifics revealed the tendency to pay forward outcomes, divorced from potential selfish motives present in zero-sum distribution tasks. In addition, using the same value reward across both options for the Actors removed any confounds related to differences in or distractions from their own outcome.

## Results

**Figure 3:**
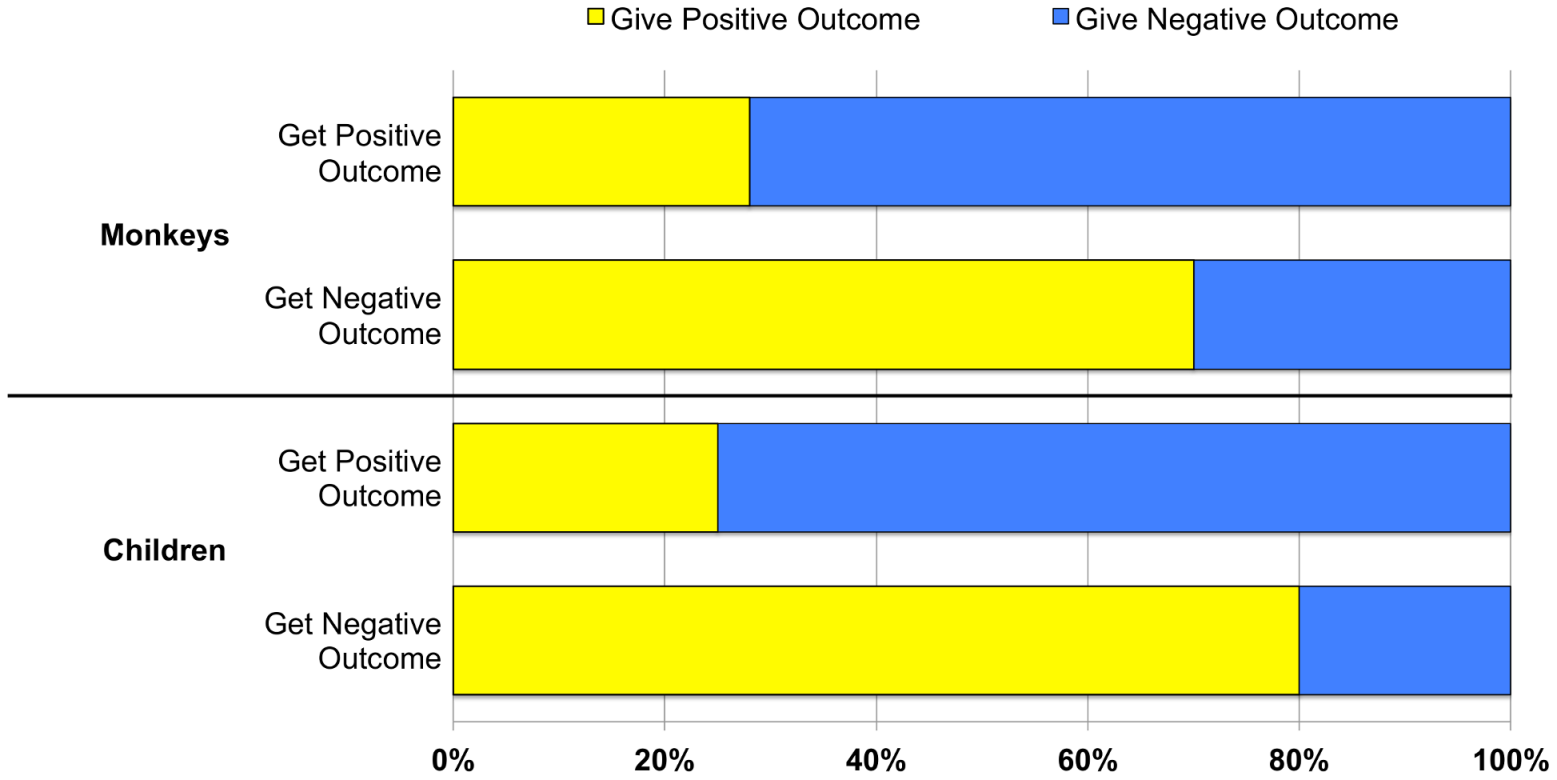
Percentage of total trials in which monkeys and children paid forward positive and negative outcomes after receiving positive and negative outcomes.

Actors’ distributions were strongly related to previously received outcomes, for both monkeys (n = 4 participants, 22 trials, Fisher’s exact, *p* = .03) and children (n = 48 children, 48 trials, Fisher’s exact, *p* = .009). Monkeys paid forward negative outcomes 75% of the time and positive outcomes 80% of the time; children paid forward negative outcomes 72% of the time and positive outcomes 70% of the time. The rates at which positive versus negative outcomes were paid forward did not significantly differ in monkeys (*X^2^*(1, N = 17) = .06, *p* = .81) or children (*X* ^2^(1, N = 34) = .12, *p* = .73). Similarly, the rates at which children versus monkeys paid forward positive (*X^2^*(1, N = 33) = .38, *p* = .54) and negative (*X^2^*(1, N = 37) = .04, *p* = .85) outcomes were not statistically different across species. See Figure 3 for all results.

We also performed a series of logistic regressions in order to confirm that the giving behavior of both children and monkeys truly reflected a tendency to pay forward both positive and negative behavior in kind. If this was the case, then previously received outcomes should predict giving behavior even when controlling for all other possible variables (e.g., identity of initial Actor, identity of Recipient). A regression on monkey giving behavior (positive, negative) using the predictors of *initial Actor identity*, *focal participant identity*, *final Receiver identity*, and *received outcome* (positive, negative) revealed that only *received outcome* affected giving behavior, Wald’s *X^2^*(1) = 7.34, *p*<.01; all other predictors *p*>.45. A regression on children’s giving behavior (positive, negative) using ‘the predictors of *initial Actor gender*, *focal participant gender*, *final Receiver gender*, and *received outcome* (positive, negative) revealed that only *received outcome* affected giving behavior, Wald’s *X^2^*(1) = 7.34, *p*<.01; all other predictors *p*>.16. Taken together, these analyses confirm that the giving behavior of both children and monkeys can be attributed to previously received outcomes–that is, they paid both positive and negative behavior forward in kind. See Tables S1 and S2 for trial-by-trial data.

## Discussion

Our results suggest that the propagation of both positive and negative behavior within social groups may stem from a mechanism that is both cognitively simple and evolutionarily old. Our finding that monkeys and 4-year-old children paid forward positive outcomes to conspecifics is line with previous behavioral findings in rats [32], and confirms that the act of paying forward positive events does not require complex emotions [15], human-specific norms [20], or sophisticated perspective-taking abilities [21]. Our results also indicate that both populations paid forward positive *and* negative outcomes, demonstrating that paying forward behavior is not limited to prosocial interactions. Instead, our findings suggest that paying forward behavior in monkeys and children is best explained by a simple give-what-you-get mechanism–one that may be the foundation upon which more complex cooperative behaviors are built in adult humans.

Overall, our findings are consistent with a contingency-based give-what-you-get strategy, a form of generalized reciprocity in which like begets like, regardless of the specific recipient or the valence of the outcome [23]. Giving what you get is less cognitively complex than other forms of reciprocity, and so is a likely explanation for group-level cooperation in non-human animals [63], [64]. Importantly, because contingency-based strategies like give-what-you-get are not sensitive to recipient identity, individuals employing them need not differentiate between paying outcomes *forward* and paying outcomes *back*; they are simply motivated to reciprocate outcomes *in kind*. As a result, this explanation implies that the pattern of behavior of monkeys and children in the current study may not necessarily be specific to paying-it-forward, but rather a reflection of a motivation to reciprocate outcomes in general. If this were the case, we would expect similar patterns of giving whether the recipient was the same or different than the individual from whom an allocation was received; that is, we would expect minimal differences between paying behavior forward and paying behavior back. However, if the mechanism underlying our results is specific to paying-it-forward, we would expect different patterns of giving when individuals have the opportunity to pay outcomes *back* to the initial actor. While the current data do not allow us to make this distinction, future research could examine the extent to which common and distinct mechanisms underlie these two related behaviors.

In our results, monkeys and children appear to pay forward like outcomes at equally high rates, despite evidence suggesting predispositions toward prosociality in other contexts [39]–[43]. The current data do not include baseline levels of giving, but a follow-up study comparing general rates of positive/negative outcome distribution with rates after participants receive a positive/negative outcome themselves would offer insight into the relative strength of the drive to pay forward positive vs. negative outcomes. Nonetheless, our findings show a clear pattern of behavior in which giving in both monkeys and children is influenced by the valence of received outcomes; these data suggest that a “give what you get” strategy drives the tendency to pay forward both positive and negative outcomes.

A second (and not mutually exclusive) possibility consistent with our results is that pay-it-forward tendencies are driven by basic affective processes, ones that may be precursors to the more sophisticated emotions observed in adult humans [10]. Whereas affect (i.e. positive and negative feelings) occur automatically [65], [66] and across species [67], gratitude is considered a secondary emotion requiring additional cognitive resources to interpret initial basic affective responses [15], [68], [69]. Capuchin monkeys do not possess the suite of cognitive abilities associated with explanations relying on complex emotions like gratitude, and thus these emotions cannot drive pay forward behaviors as some have hypothesized [15]–[18]. However, both children and capuchins possess basic affective processes that may drive paying forward of both positive and negative outcomes. Indeed, affect has been shown to motivate future behavior in adult humans [10], [31], [70]–[73] as well other primate species [74]–[76]; further research may determine the role of affective factors in pay-it-forward behaviors in capuchin monkeys and children.

While the form of paying-it-forward we observed in capuchin monkeys and young children does not require secondary emotions, perspective-taking abilities, or uniquely human social or moral norms, these factors are likely important in adult humans. Our results therefore hint that the mechanism underlying pay-it-forward behavior in children and monkeys serves as a framework around which more sophisticated social, emotional, and moral decision-making systems are built in adult humans. For example, while adult humans–like monkeys and children–surely experience basic affective responses after experiencing a positive or negative outcome, they may be uniquely be able to draw upon a more sophisticated suite of cognitive abilities with which to reflect upon their experiences and determine their future actions. Whereas monkeys and children in our study consistently paid forward negative outcomes despite the no-cost nature of the task, the general tendency to pay forward negative outcomes in adults may be overshadowed by more cognitively sophisticated processes such as cost/benefit analyses [26] or concerns about maintaining one’s positive reputation within the group [77]–[79]. Indeed, research shows that human adults often act in strikingly self-interested ways when cost/benefit analyses tip in their favor and the likelihood of negative reputational repercussions is low [80]–[82], suggesting that increased cognitive sophistication doesn’t necessitate increased prosociality, but rather facilitates flexible decision-making processes. Likewise, it is probable that paying forward generosity in human adults is not merely due to general positive affect, but is instead the result of further cost/benefit analyses, secondary emotions like gratitude [15], adherence to cultural or religious norms [19], and/or means to attain the warm glow that comes as the result of being the cause of another’s good fortune [80]. In the end, though, all of these more cognitively complex factors may merely be building on–or modifying–the simple strategies evident in the behavior of organisms such as capuchin monkeys and human children.

Our results indicate that the propagation of prosocial behavior within groups is not rooted in prosocial motives alone, but instead emerges via a simple mechanism, shared across phylogeny and ontogeny, that encourages paying forward both positive and negative behaviors in kind. Our results suggest that even the most heartwarming acts of paying forward generosity likely have their roots in a simple mechanism that is not limited to prosocial tendencies. While emotions like gratitude and uniquely human norms likely play a role in the extraordinary cases of paying forward generosity that make newspaper headlines, our data suggest paying-it-forward may propagate and persist within social groups, even in the absence of these factors. Although a fascination with the propagation of kindness–and a tendency to explain these behaviors in moralistic terms–may be uniquely human, the mechanism underlying this behavior is likely not.

## Supporting Information

Table S1 Positive/Negative outcome distribution patterns within testing chains for monkeys. Each row represents a discrete test session; monkeys only participated in one test session per day. Trials in which monkeys ‘gave what they got’ are bolded. Trials in which monkeys paid forward negative outcomes are highlighted in blue; trials in which monkeys paid forward positive outcomes are highlighted in yellow.
(TIF)

Table S2 Positive/Negative outcome distribution patterns within testing chains for children. Each row represents a single testing chain; variance in chain length is due to variance in the number of consenting children per classroom. Trials in which children (males = M; females = F) ‘gave what they got’ are bolded. Trials in which children paid forward negative outcomes are highlighted in blue; trials in which children paid forward positive outcomes are highlighted in yellow.
(TIF)

Methods S1 Supplemental methods.
(DOCX)
