# Correction: Give What You Get: Capuchin Monkeys (*Cebus apella*) and 4-Year-Old Children Pay Forward Positive and Negative Outcomes to Conspecifics

**DOI:** 10.1371/journal.pone.0096959

**Published:** 2014-04-29

**Authors:** The PLOS ONE Staff

Figure 3 is incorrect. The authors have provided a corrected version here.

**Figure 3:**
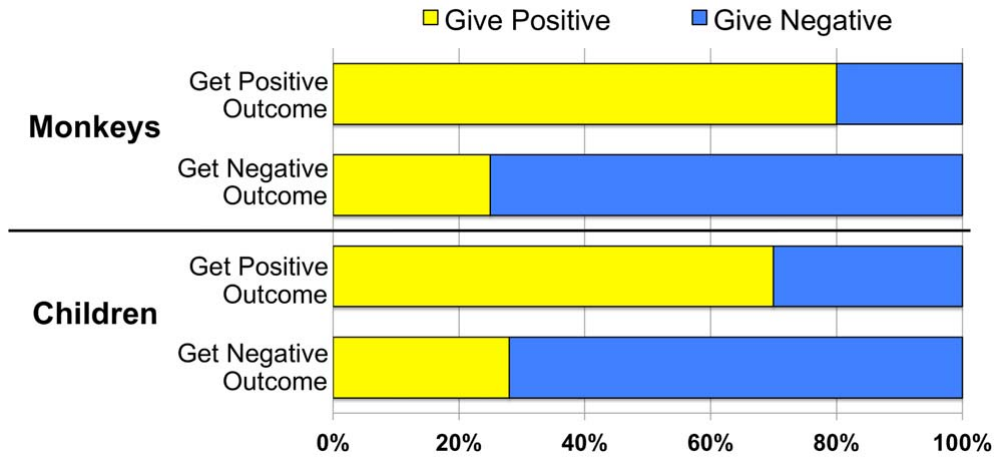
Percentage of total trials in which monkeys and children paid forward positive and negative outcomes after receiving positive and negative outcomes.
