# Supplementary figures and images for: Give What You Get: Capuchin Monkeys (Cebus apella) and 4-Year-Old Children Pay Forward Positive and Negative Outcomes to Conspecifics
Source: PLoS One. 2014 Jan 29;9(1):e87035. doi: 10.1371/journal.pone.0087035 (PMC3906089; doi:10.1371/journal.pone.0087035)

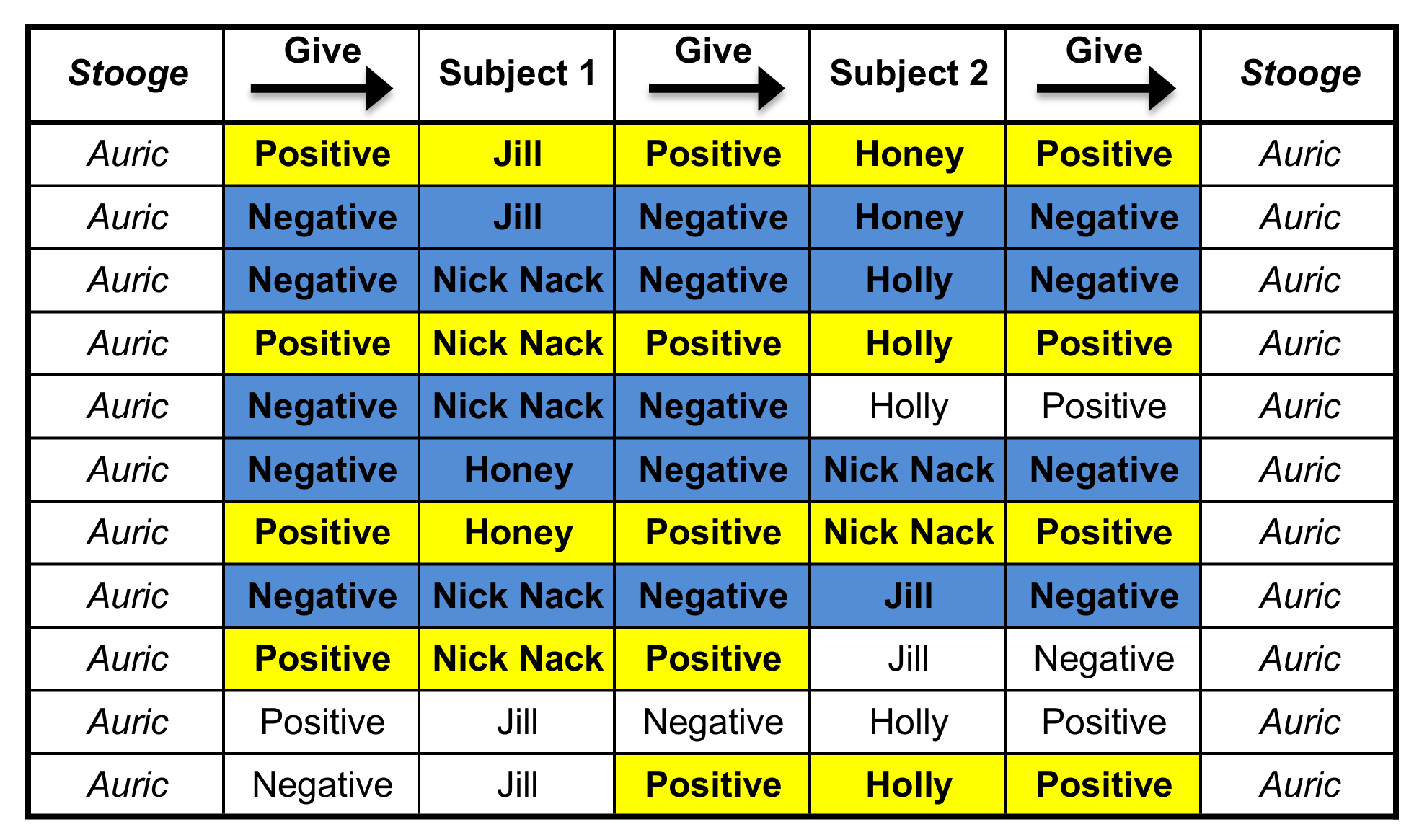

Supplement: Table S1 — Positive/Negative outcome distribution patterns within testing chains for monkeys. Each row represents a discrete test session; monkeys only participated in one test session per day. Trials in which monkeys ‘gave what they got’ are bolded. Trials in which monkeys paid forward negative outcomes are highlighted in blue; trials in which monkeys paid forward positive outcomes are highlighted in yellow. (TIF) [file pone.0087035.s001.tif]

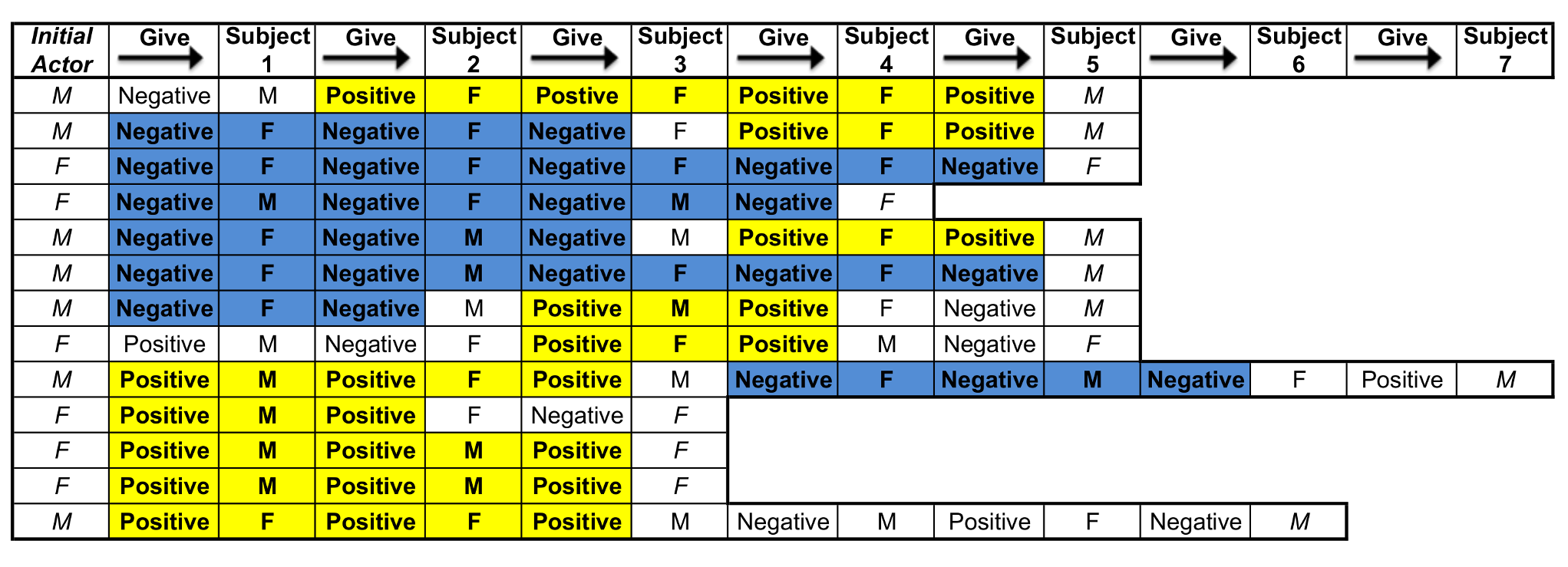

Supplement: Table S2 — Positive/Negative outcome distribution patterns within testing chains for children. Each row represents a single testing chain; variance in chain length is due to variance in the number of consenting children per classroom. Trials in which children (males = M; females = F) ‘gave what they got’ are bolded. Trials in which children paid forward negative outcomes are highlighted in blue; trials in which children paid forward positive outcomes are highlighted in yellow. (TIF) [file pone.0087035.s002.tif]
