# Supplementary material for: Give What You Get: Capuchin Monkeys (Cebus apella) and 4-Year-Old Children Pay Forward Positive and Negative Outcomes to Conspecifics
Source: PLoS One. 2014 Jan 29;9(1):e87035. doi: 10.1371/journal.pone.0087035 (PMC3906089; doi:10.1371/journal.pone.0087035)
Supplement: Methods S1 — Supplemental methods. (DOCX) [file pone.0087035.s003.docx]

**Supporting Information**

**Monkeys.** All monkey participants had extensive experience with the testing apparatus from a previous study. Similar to the testing protocol used for children, monkeys were tested in chains of participants, such that individuals first received from, and then gave to, a familiar conspecific. Unlike the children who were randomly assigned a position in the testing chain, monkeys were tested in predetermined groups of three with the first and last monkey in the chain (AG) being a trained Stooge. The Stooge was trained to pull only the left lever of the apparatus, thus allowing experimenters the flexibility to arrange allocations in the apparatus such that it appeared AG was willingly delivering positive or negative outcomes. The use of a Stooge maximized data collection within a limited sample size by allowing experimenters to control the outcome distributed on the first trial within each chain. AG was chosen to be the Stooge monkey because he had previous experience with the apparatus and was also the lowest ranking monkey in the group, thus controlling for any influence relative rank may have played on the initial or final distribution in each testing chain.

The order in which monkeys were placed within testing chains was determined prior to the commencement of testing and was dictated by the need to control for several social factors. First, precautions were taken to minimize the possibility of task-specific long-term reciprocation by ensuring that a given monkey never served as an Actor for another monkey from whom they had previously received an allocation over the course of testing. However, due to the small number of monkeys in our colony and the nature of the testing paradigm, we were not able to control for direction of the interactions with the Stooge monkey. While participants both received from, and gave to, the Stooge monkey over the course of the study, they never did so within the same day and there were often several days between test sessions for each participant. Second, efforts were made to control for inter-dyad relationship quality by ensuring that each monkey received from, and gave to, at least 2 different monkeys over the course of testing. Additionally, no matrilineal dyads were tested together, meaning female participants never gave to, or received from, their offspring. Finally, monkeys only participated in one test session per day (and, thus only received and gave one distribution per day), with an average of 8.35 ± 2.3 days between test sessions for each participant in an effort to minimize the possibility for carry-over effects between test sessions. As a result of these criteria, some monkeys participated in more test sessions than others; specifically, subject NN participated in 7 test sessions, subject JM participated in 6 test sessions, subject HG participated in 5 test sessions, and subject HR participated in 4 test sessions. See Table S1 for a detailed table illustrating all monkey testing chains and individual outcomes.

Monkeys were tested in enclosures that were spatially and visually distant from the rest of the social group. Each monkey was tested in his/her own enclosure (71 cm^3^), with the apparatus situated between the two enclosures such that both monkeys could see the allocation distributions and one another over the top of it. Testing began when the Stooge monkey “chose” to deliver either a positive or a negative outcome to the first participant (Subject 1). After collecting their respective food allocations, the Stooge left the testing area and Subject 1 moved into his testing enclosure to assume the role of Actor. A third monkey (Subject 2)—unaware of the outcome of the previous interaction—then entered the enclosure vacated by Subject 1 and assumed the role of Recipient. Subject 1 was then given the option to deliver a positive or negative outcome to Subject 2. After collecting their respective food allocations, Subject 1 left the testing area, Subject 2 moved into her testing enclosure to assume the role of Actor, and the Stooge—unaware of the outcome of the previous interaction—reentered the testing area to assume the role of Recipient for Subject 2. After Subject 2 delivered a positive or negative outcome to the Stooge monkey, the test session was complete and all monkeys were reunited with the social group. With the exception of the Stooge monkey, all monkeys were first a Recipient and then an Actor, creating a chain of allocations where each monkey received *from* and gave *to* different monkeys from their social group.

The first distribution of each testing chain was counterbalanced such that half of the testing chains began with AG distributing a positive outcome and half of them began with a negative outcome; the order in which positive/negative distributions were presented on the first trial was randomized. Finally, the placement of the distribution options (positive/negative) in the apparatus for all Actors was counterbalanced such that each option was presented on the left side of the box 50% of the time and the right side of the box 50% of the time.

**Children***.*  Prior to the commencement of testing, children were brought into the testing area from their classrooms as a group to receive training on the operation of the apparatus and an explanation of the testing procedure. A previous study [Leimgruber, Shaw, Santos & Olson, 2012] showed that children found the operation of the apparatus to be quite intuitive, and the present study proved no different. The training procedure consisted of a demonstration of the mechanics of the apparatus using colored bouncy balls (i.e. “If you pull the lever on the left you will get what is here [point] and you will deliver what is here [point] to whomever is across the table from you.”), followed by a series of hypothetical questions regarding the outcomes (i.e. “If I pull this lever, what color ball will I get? Which lever do I pull to deliver a red ball to the person sitting on the other side of the table?”). Once it was confirmed that children understood the apparatus, they were told that they would have a chance to use the apparatus to distribute stickers to themselves and their classmates.

Children were given the opportunity to ask clarifying questions as a group before the experimenters randomly selected two children to remain in the testing area with Experimenter 1. The remainder of each group was taken out of visual and audio contact by Experimenter 2 who kept the children waiting to participate occupied with a distracting task (e.g. reading a book, doing a puzzle, playing with blocks). Tasks were dependent on readily available school materials and were intended to discourage discussion of the experiment between participants. One of the two participants remaining in the testing area was randomly designated the Actor and the other was designated the role of Recipient. The Actor was then given the choice between distributing a positive or negative outcome to the Recipient, and the Recipient received whatever she was given. Once the Actor had made her distribution decision and both children had collected their allocations, this initial Actor left the testing area to join the other children and the original Recipient assumed the new role of Actor. A third child, unaware of what had happened on the previous trial, entered the room to assume the role of the new Recipient and the procedure described above was repeated. This chain of events continued until all children had the opportunity to first receive from, and then give to, a classmate. Similar to the procedure used with monkeys, the original Actor served as a Recipient for the last child in the chain. See Table S2 for detailed table illustrating testing chains and individual outcomes for children. Once all children had participated in the study, they returned to their classroom as a group. With the exception of the first Actor, all children assumed the role of Recipient first and Actor second, thus creating a chain of allocations where each child received *from* and gave *to* different peers from their social group.

The placement of the distribution options (positive/negative) into the apparatus was varied within test sessions, such that the lever associated with positive/negative outcomes alternated between participants. As a result, a participant who received a positive outcome via the lever on the north-facing side of the apparatus would have to pull the lever on the south-facing side of the apparatus to deliver a positive outcome to a third conspecific.

**Data Collection**. All monkey test sessions were videotaped and experimenters recorded choice data was recorded live during testing; choice data was later confirmed from video by a second coder. Children’s test sessions were not videotaped and choice data was recorded live during testing. The authors would like to note that a subset of the monkey testing videos (n=6) are no longer available due to a technical error that occurred while transferring data.
